# Exploiting the Concept of Multivalency with ^68^Ga- and ^89^Zr-Labelled Fusarinine C-Minigastrin Bioconjugates for Targeting CCK2R Expression

**DOI:** 10.1155/2018/3171794

**Published:** 2018-04-10

**Authors:** Dominik Summer, Christine Rangger, Maximilian Klingler, Peter Laverman, Gerben M. Franssen, Beatrix E. Lechner, Thomas Orasch, Hubertus Haas, Elisabeth von Guggenberg, Clemens Decristoforo

**Affiliations:** ^1^Department of Nuclear Medicine, Medical University Innsbruck, Anichstrasse 35, 6020 Innsbruck, Austria; ^2^Department of Radiology & Nuclear Medicine, Radboud University Medical Center, Geert Grooteplein Zuid 10, 6525 GA Nijmegen, Netherlands; ^3^Division of Molecular Biology, Medical University Innsbruck, Innrain 80/82, 6020 Innsbruck, Austria

## Abstract

Cholecystokinin-2 receptors (CCK2R) are overexpressed in a variety of malignant diseases and therefore have gained certain attention for peptide receptor radionuclide imaging. Among extensive approaches to improve pharmacokinetics and metabolic stability of minigastrin (MG) based radioligands, the concept of multivalency for enhanced tumour targeting has not been investigated extensively. We therefore utilized fusarinine C (FSC) as chelating scaffold for novel mono-, di-, and trimeric bioconjugates for targeting CCK2R expression. FSC-based imaging probes were radiolabelled with positron emitting radionuclides (gallium-68 and zirconium-89) and characterized* in vitro* (log⁡*D*, IC_50_, and cell uptake) and* in vivo* (metabolic stability in BALB/c mice, biodistribution profile, and microPET/CT imaging in A431-CCK2R/A431-mock tumour xenografted BALB/c nude mice). Improved targeting did not fully correlate with the grade of multimerization. The divalent probe showed higher receptor affinity and increased CCK2R mediated cell uptake while the trimer remained comparable to the monomer.* In vivo* biodistribution studies 1 h after administration of the ^68^Ga-labelled radioligands confirmed this trend, but imaging at late time point (24 h) with ^89^Zr-labelled counterparts showed a clearly enhanced imaging contrast of the trimeric probe compared to the mono- and dimer. Furthermore,* in vivo* stability studies showed a higher metabolic stability for multimeric probes compared to the monomeric bioconjugate. In summary, we could show that FSC can be utilized as suitable scaffold for novel mono- and multivalent imaging probes for CCK2R-related malignancies with partly improved targeting properties for multivalent conjugates. The increased tumour accumulation of the trimer 24 h postinjection (p.i.) can be explained by slower clearance and increased metabolic stability of multimeric conjugates.

## 1. Introduction

**Scheme 1:**
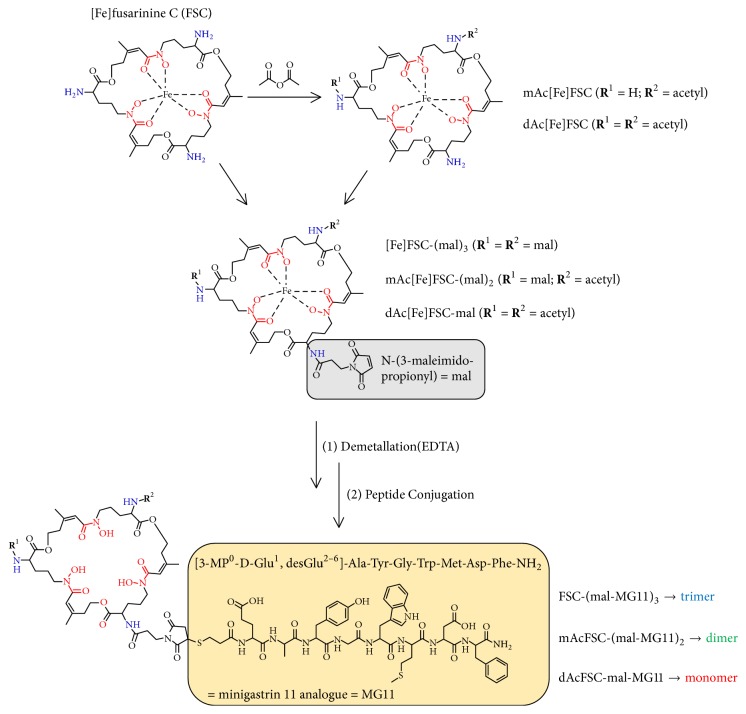
Route of synthesis for novel mono- and multimeric MG conjugates (stereochemistry omitted).

Receptor targeting with radiolabelled peptides has become an emerging field in nuclear medicine for early diagnosis and therapy of cancerous diseases [1, 2]. The overexpression of cholecystokinin receptor subtype 2 (CCK2R) is involved in various malignancies, such as medullary thyroid carcinoma (MTC), small cell lung cancer (SCLC), and neuroendocrine tumours (NET) [3, 4] and therefore represents an interesting target for peptide receptor radionuclide imaging and therapy. In a preclinical study, different derivatives based on human minigastrin (H_2_N-Leu-(Glu)_5_-Ala-Tyr-Gly-Trp-Met-Asp-Phe-NH_2_) for targeting CCK2R expression were investigated in order to find the ideal targeting sequence [5]. This study reported that four* C*-terminal amino acids (-Trp-Met-Asp-Phe-NH_2_) are mandatory for selectivity and high affinity towards CCK2R. Different variations are allowed in the* N*-terminal region to improve* in vivo* targeting properties and pharmacokinetics, especially kidney uptake which is closely related to the appearance of negatively charged amino acids. Based on this knowledge a variety of MG derivatives have been synthesized by different groups with the aim of reducing kidney but retaining tumour uptake [6]. Further studies have shown that these modifications are unfortunately accompanied by low metabolic stability predominantly for MG11 [7, 8].* In vivo* stability is a major issue for imaging of receptor expression with small peptide-based molecules. Rapid degradation may lead to decreased tumour uptake and low imaging contrast. Several attempts have been made to overcome this issue, for example, coinjection of the neutral endopeptidase inhibitor phosphoramidon [9] with promising results. Less effort has been spent on a different approach: the design of multivalent constructs. This could increase the probability of receptor target interaction and therefore increase receptor avidity [10, 11] as well as promoting the formation of metabolites able to rebind to the receptor. By this, increased apparent stability tumour uptake and therefore improved imaging contrast would be achieved as has been proposed by Carlucci and coworkers [12]. Sosabowski and coworkers were, to the best of our knowledge, the only group so far reporting on a divalent probe (MGD5) for nuclear imaging of CCK2R expression. This tracer consisting of the bifunctional chelator (1,4,7,10-tetraazacyclododecane-1,4,7,10-tetraacetic acid, DOTA) conjugated to a MG derivative crosslinked via thiol-maleimide to a second sequence of the peptide was radiolabelled with indium-111 for single photon emission tomography (SPECT). It showed increased affinity and tumour uptake compared to its monomeric counterpart ^111^In-APH070 [13]. Our group recently reported on fusarinine C (FSC), a cyclic siderophore based bifunctional chelator, providing a scaffold for site-specific conjugation of up to three targeting vectors with excellent complexing properties towards the PET radionuclides gallium-68 and zirconium-89 [14, 15]. As positron emission tomography (PET) provides higher resolution compared to SPECT we initiated this study to compare novel mono-, di-, and trimeric FSC-conjugates for targeting CCK2R expression. Due to low metabolic stability minigastrin analogue (MG11) was chosen as model peptide and was conjugated via thiol-maleimide crosslink as shown in Scheme 1. The resulting mono- and multimeric conjugates were radiolabelled with gallium-68 and zirconium-89 followed by* in vitro* and* in vivo* characterization.

## 2. Experimental Section

### 2.1. Analytical [Radio]-RP-HPLC

Reversed-phase (RP) high-performance liquid chromatography (HPLC) analysis was carried out using the following instrumentation: UltiMate 3000 RS UHPLC pump, UltiMate 3000 autosampler, UltiMate 3000 column compartment (25°C oven temperature), UltiMate 3000 Variable Wavelength Detector (Dionex, Germering, Germany; UV detection at *λ* = 220 nm), radio-detector (Gabi Star, Raytest; Straubenhardt, Germany), Jupiter 5 *μ*m C_18_ 300 Å 150 × 4.6 mm (Phenomenex Ltd. Aschaffenburg, Germany) column with acetonitrile (ACN)/H_2_O/0.1% trifluoroacetic acid (TFA) as mobile phase; flow rate of 1 mL/min; gradient: 0.0–3.0 min 0% ACN, 3.0–5.0 min 0–30% ACN, 5.0–20.0 min 30–60% ACN, 20.0–25.0 min 60–80% ACN.

### 2.2. Preparative RP-HPLC

Sample purification via RP-HPLC was performed on a Gilson 322 Pump with a Gilson UV/VIS-155 detector (UV detection at *λ* = 220 nm) using a PrepFC™ automatic fraction collector (Gilson, Middleton, WI, USA). Following ACN/H_2_O/0.1% TFA multistep gradients were used on a Eurosil Bioselect Vertex Plus 30 × 8 mm 5 *μ*m C_18_A 300 Å precolumn and Eurosil Bioselect Vertex Plus 300 × 8 mm 5 *μ*m C_18_A 300 Å column (Knauer, Berlin, Germany) and a flow rate of 2 mL/min:* gradient A*: 0.0–1.0 min 10% ACN, 1.0–25.0 min 10–50% ACN, 25.0–28.0 min 50% ACN, 28.0–30.0 min 10% ACN;* gradient B*: 0.0–1.0 min 20% ACN, 1.0–26.0 min 20–60% ACN, 26.0–28.0 min 60% ACN, 28.0–30.0 min 60–20% ACN;* gradient C*: 0.0–1.0 min 20% ACN, 1.0–26.0 min 20–80% ACN, 26.0–28.0 min 80% ACN, 28.0–30.0 min 20% CAN.

### 2.3. MALDI-TOF MS

Matrix-assisted laser desorption/ionization time-of-flight mass spectrometry was performed on a Bruker microflex™ bench-top MALDI-TOF MS (Bruker Daltonics, Bremen, Germany). Samples were prepared on a microscout target (MSP96 target ground steel BC, Bruker Daltonics) using dried-droplet method and *α*-cyano-4-hydroxycinnamic acid (HCCA, Sigma-Aldrich, Handels GmbH, Vienna, Austria) as matrix. All spectra were recorded by summarizing 800 laser shots per spot and Flex Analysis 2.4 software was used for data processing.

### 2.4. Radio-ITLC

Instant thin layer chromatography (ITLC) analysis was performed using TLC-SG strips (Varian, Lake Forest, CA, USA) and 0.1 M sodium citrate solution (pH 5) for ^68^Ga-conjugates and 0.05 M EDTA solution (pH 7) for ^89^Zr-conjugates as mobile phase. The strips were analyzed using a TLC scanner (Scan-RAM™, LabLogistic, Sheffield, UK). Radiolabelled bioconjugates remained at the start (*R*_*f*_ = < 0.1) while free radionuclides migrated with the solvent front (*R*_*f*_ = > 0.9).

### 2.5. Gamma Counter

A 2480 Automatic Gamma Counter Wizard2 3′′ (Perkin Elmer, Waltham, MA, USA) was used to measure the radioactivity of samples retrieved from* in vitro* and* in vivo* experiments.

### 2.6. Cell Culture

The human epidermoid carcinoma cell line (A431) stably transfected with the plasmid pCR3.1 containing the full coding sequence for the human CCK2R (A431-CCK2R) and with the empty vector alone (A431-mock) was a kind gift from Aloj [16]. Both cell lines were maintained at 37°C in a humidified atmosphere of 95% air/5% carbon dioxide in tissue culture flasks (Cellstar; Greiner Bio-One, Kremsmunster, Austria) using Dulbecco's Modified Eagle's Medium (DMEM) supplemented with 10% v/v fetal bovine serum (FBS) and 1% v/v penicillin-streptomycin-glutamine (PSG) solution media (Gibco, Invitrogen Corporation, Paisley, UK).

### 2.7. Precursor Preparation

The synthesis of mono- and multivalent FSC-based minigastrin derivatives is presented in detail in the supplementary materials sections.

### 2.8. Radiochemistry

#### 2.8.1. Radiolabelling with Gallium-68

Fractionated elution of a commercially available ^68^Ga/^68^Ge-generator (IGG100, Eckert & Ziegler Isotope Products, Berlin, Germany; nominal activity of 1850 MBq) with 0.1 M hydrochloric acid (HCl, Rotem Industries, Israel) was used to obtain ^68^GaCl_3_ (gallium chloride, ~310 MBq) in 1 mL eluate. For labelling 20 *μ*g (5–10 nmol) of conjugate (mono-, di-, or trimer) was mixed with 100–500 *μ*L eluate (~30–160 MBq) and the pH was adjusted to 4.5 by adding 20 *μ*L of sodium acetate solution (1.14 M) per 100 *μ*L eluate. The mixture was incubated for 5–15 min at RT and then analyzed by* radio*-ITLC and* radio*-RP-HPLC. Following this procedure but using a 100-fold molar excess of GaBr_3_ (gallium bromide) dissolved in 0.1 N HCl instead of generator eluate gave the ^nat^Ga-chelated peptides which were used for binding affinity measurements.

#### 2.8.2. Radiolabelling with Zirconium-89

The cyclotron produced radionuclide was purchased from PerkinElmer (Waltham, US) and delivered as ^89^Zr-oxalic acid solution (1 M and ~1 MBq/*μ*L). Approximately 10 MBq (10 *μ*L) was neutralized with 9.6 *μ*L sodium carbonate solution (1 M) at RT. After 3 min 100 *μ*L of 4-(2-hydroxyethyl)-1-piperazineethanesulfonic acid (HEPES) buffer (0.5 M, pH 6.98) was mixed with the radionuclide solution followed by addition of the corresponding peptide (20 *μ*g) and the mixture was incubated for 30–60 min at ambient temperature. The reaction was monitored by* radio*-ITLC. In case of animal experiments 20 *μ*L of CaCl_2_ (calcium chloride) was added to precipitate CaC_2_O_4_ (calcium oxalate). The resulting suspension was centrifuged for 5 min at 14 × 10^3^ rpm (Eppendorf Centrifuge 5424, Hamburg, Germany) and an aliquot from the supernatant was further diluted in PBS for* in vivo* evaluation. Representative chromatograms are shown in the Supplementary Materials (Figure S1).

### 2.9. *In Vitro* Characterization

#### 2.9.1. Stability Study

To assess the stability of the radionuclide complex the radiolabelled peptides were diluted with PBS to a concentration of 5 *μ*M. Aliquots of 50 *μ*L were mixed with PBS (as control), human serum, and a 1.000-fold molar excess over radioligand of either EDTA (pH 7), DTPA (pH 7), or FeCl_3_ (pH 5). Then samples were incubated in duplicate at 37°C up to 4 h for gallium-68 and up to 7 days for zirconium-89 labelled peptides. Samples were analyzed at selected time points via radio-RP-HPLC for ^68^Ga-labelled probes and radio-ITLC for ^89^Zr-labelled peptides. The ITLC strips were cut into half and measured in the gamma counter to determine the percentage of labelled peptide (origin) to free radionuclide (solvent front).

#### 2.9.2. Distribution Coefficient (log⁡*D*)

Aliquots (50 *μ*L) of ^68^Ga- and ^89^Zr-labelled mono-, di-, and trimer (10 *μ*M) were diluted in 450 *μ*L PBS. After adding 500 *μ*L octanol the mixture was vortexed with 1400 rpm (MS 3 basic vortexer, IKA, Staufen, Germany) for 15 min at RT followed by centrifugation for 2 min at 4500 rpm. Subsequently, 100 *μ*L aliquots of the organic and the aqueous layer were collected and log⁡*D* values were calculated using Excel (*n* = 3, six replicates) after gamma counter measurement.

#### 2.9.3. Protein Binding

For protein binding measurement ^68^Ga- and ^89^Zr-labelled peptides were incubated in PBS as control and fresh human serum and samples were maintained at 37°C. After 1, 2, and 4 h aliquots (25 *μ*L) were analyzed by size exclusion chromatography using MicroSpin G-50 columns (Sephadex G-50, GE Healthcare, Vienna, Austria) according to manufacturer's protocol. The samples were measured in the gamma counter and the percentage between protein-bound (eluate) and free conjugate (column) was calculated.

#### 2.9.4. Whole Cell Receptor Binding Affinity Studies (IC_50_)

A431-CCK2R cells were diluted to a density of 5 × 10^6^ cells/mL in 50 mM HEPES buffer (pH 7.3) containing 5 mM MgCl_2_ (magnesium chloride) and 0.3% bovine serum albumin (BSA). MultiScreen Filter Plates HTS (96-wells, 1 *μ*m glass fiber filter, Merck Millipore, Darmstadt, Germany) were washed twice with 200 *μ*L of 10 mM TRIS-buffered saline (pH 7.3) and 100 *μ*L of cell suspension was added to each well. Hereafter cells were incubated in triplicate with increasing concentrations [0.001−1.000 nM] of competitor solution (50 *μ*L of metal-bound ([^nat^Ga]) mono-, di-, and trimer as well as DOTA- MG11 as reference diluted in 20 mM HEPES, 10 mM MgCl_2_ and 0.1% BSA). After 10 min incubation at RT 50 *μ*L of radioligand (human ^125^I-[Leu^15^]-Gastrin I, 4.5 × 10^4^ cpm, prepared as previously published [17]) was added and the plate was maintained under shaking conditions (Compact Shaker KS-15 control, 200/min) for 1 h. Thereafter each well was washed twice with 200 *μ*L TRIS-buffered saline. Then the filters were measured in the gamma counter and IC_50_ values were calculated by using nonlinear curve fitting with Origin 6.1 software (Northampton, MA, USA) according to the following formula: [NS + SB/(1 + *x*/IC50)].

#### 2.9.5. CCK-2 Receptor Internalization Assay

Determination of the receptor-mediated radioligand uptake in A431- CCK2R cells was conducted as previously published [17]. Briefly, 2 × 10^6^ cells were seeded per well (12-well plates, Nunc, Thermo Scientific) and left to grow over night. After washing twice and adjusting the volume to 1.2 mL with DMEM containing 1% FBS, 150 *μ*L blocking solution (100-fold molar excess of pentagastrin in PBS/0.5% BSA solution) was added to one-half while 150 *μ*L PBS/0.5% BSA was added to the other half of wells for volume compensation. Hereafter, 150 *μ*L of the radiolabelled conjugate (diluted in PBS/0.5% BSA, approximately 30.000 cpm) was added to have a final concentration of approximately 1 nM in the assay and the plates were incubated at 37°C. After 1 h, 2 h, and 4 h (in case of zirconium-89) the cells were washed with ice-cold medium (=wash fraction), with ice-cold 0.05 M glycine buffer (pH 2.8) (membrane bound fraction) and finally lysed with 2 M sodium hydroxide (internalized fraction). All fractions were measured in the gamma counter to determine the percentage of cell associated radioactivity in relation to total activity added.

### 2.10. *In Vivo* Characterization

All animal experiments were conducted in compliance with the Austrian and Dutch animal protection laws and with approval of the Austrian Ministry of Science (BMWF-66.011/000604-II/3b/2012 and BMWFW-66.011/0049-WF/II/3b/2014).* In vivo* stability studies were conducted in 5-week-old female BALB/c mice (in-house breed, ZVTA Innsbruck, Dr. Hermann Dietrich). The biodistribution studies were performed using female 8−10-week-old athymic BALB/c nude mice (Charles River Laboratories, Sulzfeld, Germany). Tumour xenografts were induced by subcutaneous injection of 2 × 10^6^ A431-CCK2R cells (receptor positive) in the right and the same amount of A431-mock (receptor negative) in the left flank. Tumours were allowed to grow until they had reached a volume of 0.3–0.6 cm^3^.

#### 2.10.1. *In Vivo* Stability

^68^Ga-labelled conjugates were injected via a lateral tail vein using an amount of 1.5 nmol and a radioactivity of 15 MBq. After 5 min mice were euthanized by cervical dislocation and blood samples were taken by heart puncture. Aliquots of the blood were immediately precipitated by adding 0.1% TFA/ACN (1 : 1 v/v) and the supernatant was diluted with H_2_O and analyzed by radio-RP-HPLC.

#### 2.10.2. Ex Vivo Biodistribution

To evaluate the biodistribution profile nude mice (*n* = 4) were intravenously injected with either 1-2 MBq ^68^Ga- or 0.3 MBq ^89^Zr-labelled mono-, di-, and trimer (0.1–0.2 nmol). The animals were sacrificed by cervical dislocation after 1 h as well as 2 h and 4 h in case of ^68^Ga- and ^89^Zr-labelled trimer, followed by the collection of organs and tissue. The collected samples were measured in the gamma counter and the results were calculated as percentage of injected dose per gram tissue (% ID/g).

#### 2.10.3. MicroPET/CT Imaging

Small animal imaging experiments were carried out with an Inveon microPET/CT scanner (Siemens Preclinical Solutions, Knoxville, USA). A group of eight double tumour xenografted mice were injected intravenously with either 8–13 MBq of ^68^Ga-labelled tracers (0.2–0.5 nmol) or 3–5 MBq of ^89^Zr-labelled peptides (0.4–1.0 nmol). MicroPET images were acquired under general anaesthesia (isoflurane/O_2_) for 15–60 min with static PET/CT scans after 1 h and additionally after 4 and 24 h for ^89^Zr-labelled conjugates. The microPET/CT scans were reconstructed with OSEM3D-SPMAP (PET, matrix size 256 × 256) and Feldkamp (CT, Shepp Logan filter).

### 2.11. Statistical Analysis

Statistical analysis was performed using the Student's *t*-test with *P* value < 0.05 indicating significance.

## 3. Results

### 3.1. Precursor Synthesis

MG11-SH could be obtained in good yield following SPPS protocol. [Fe]FSC could be extracted from fungal culture in sufficient purity to be used for further modification without additional purification. Acetylation reaction resulted in a mixture of mono- and multiple acetylated derivatives of [Fe]FSC due to three identical primary amines but the desired products were easily accessible via preparative RP-HPLC purification. Functionalization with maleimide linker was straightforward utilizing a NHS-ester strategy and conjugation of up to three targeting vectors was conducted site-specifically via maleimide-thiol crosslink reaction. All intermediates as well as final conjugates were obtained in good yield, with excellent chemical purity (>95%; analytical RP-HPLC, UV absorption at *λ* = 220 nm), and corresponding mass analysis was in good agreement with the calculated values.

### 3.2. Radiolabelling

Mono- and multimeric conjugates were quantitatively labelled with gallium-68 after 5–15 min and after 30–60 min with zirconium-89 at high molar activities and used without further purification.

### 3.3. *In Vitro* Characterization

The complex stability was evaluated by incubating the radiolabelled conjugates in 1000-fold molar excess of EDTA, DTPA, FeCl_3_ (iron chloride), fresh human serum, and PBS over a period of 4 h for gallium-68 and 7 days for zirconium-89, respectively. The radiolabelled peptides showed no significant release of radionuclide in all media except EDTA after 7 d where 20 to 30% release of radionuclide was observed indicating excellent stability of ^68^Ga and ^89^Zr-FSC. The data from this transchelation study are presented in detail in the Supplementary Materials (Table S1).

Distribution coefficient (log⁡*D*) values and protein binding data are summarized in Table 1. The results indicate a hydrophilic character of all conjugates but, as expected, clearly showed that the grade of multimerization is accompanied by increased lipophilicity. Binding to serum proteins was low (<10%) for monomeric, moderate (10–25%) for dimeric, and high for (30–50%) trimeric conjugates. Furthermore, protein binding was consistent over time for ^68^Ga-labelled mono- and dimer but increased for ^68^Ga-trimer whereas all ^89^Zr-labelled counterparts showed a slight increase over a period of 4 h.

**Figure 1:**
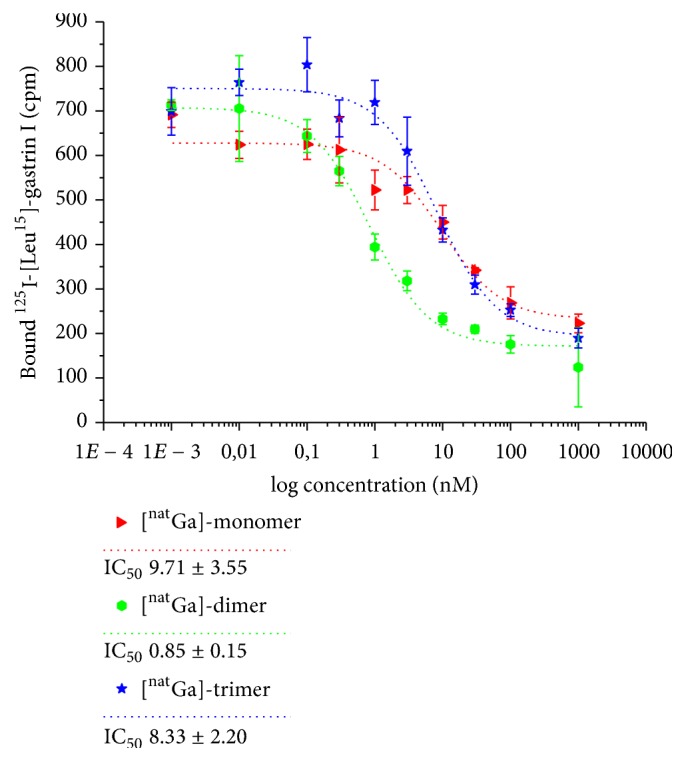
Binding affinity (IC_50_ values) of metal-bound (^nat^Ga) mono-, di-, and trimer on whole A431-CCK2R cells.

Competition assays on whole A431-CCK2R (Figure 1) revealed high binding affinity as the IC_50_ values were in the low nanomolar range for all bioconjugates. The affinity of the dimer (0.85 ± 0.15 nM) was approximately 10-fold higher while the monomer (9.7 ± 3.5 nM) and the trimer (8.3 ± 2.2 nM) remained comparable to the reference peptide DOTA-MG11 (9.5 ± 0.5 nM) [17].

**Figure 2:**
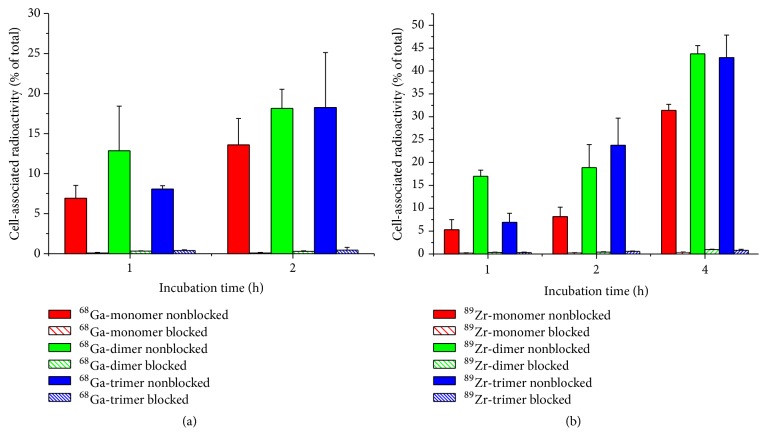
Cell-uptake studies of A431-CCK2R cells incubated with ^68^Ga-labelled mono- and multimers (a) and ^89^Zr-labelled counterparts (b). Blocking was performed with pentagastrin in 100-fold molar excess over the conjugate.

Cellular processing of A431-CCK2R cells incubated with ^68^Ga- and ^89^Zr-labelled mono- and multimeric tracers is summarized in Figure 2. In general, all conjugates showed increasing uptake over time while the unspecific cell bound fraction of corresponding blocking studies remained <1%, thus indicating highly specific receptor-mediated cell uptake. Furthermore the ^68^Ga-dimer showed significantly increased uptake after 1 h-incubation compared to the monomer (*P* = 9.01 × 10^−6^) and the trimer (*P* = 0.009) while after 2 h-incubation only the uptake of the monomer remained lower (*P* = 0.001). This trend was observed in the same manner for the ^89^Zr-labelled counterparts and was substantiated by the results after 4 h of incubation.

### 3.4. *In Vivo* Characterization

**Figure 3:**
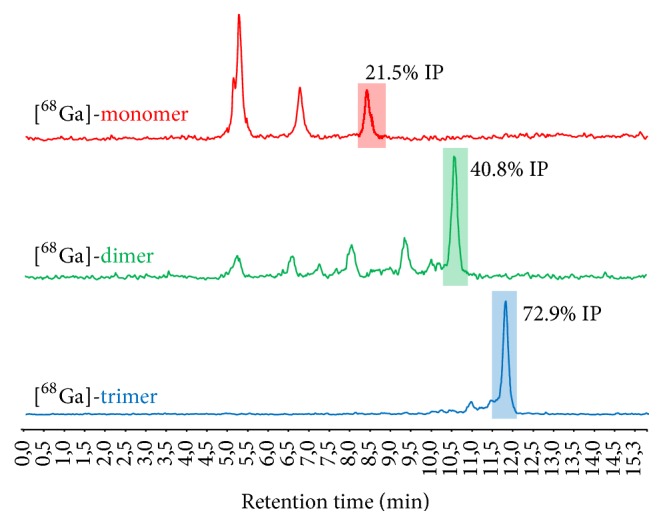
Representative* radio*-RP-HPLC chromatograms of* in vivo* stability studies in BALB/c mice 5 min p.i. (IP: intact peptide).

**Figure 4:**
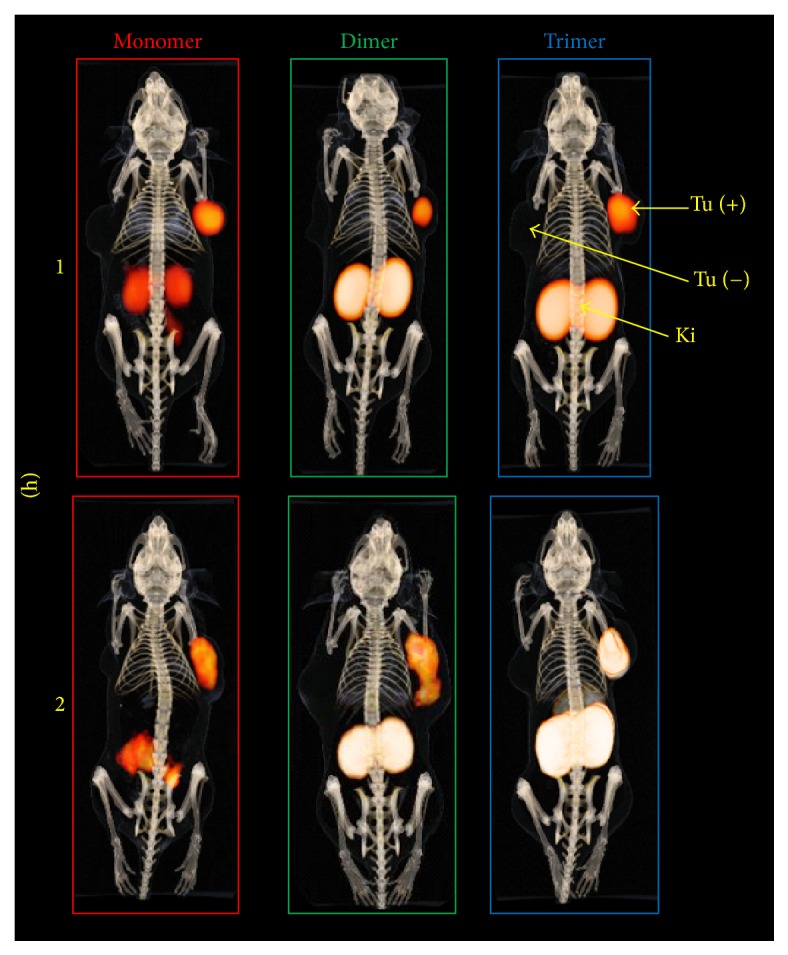
Three-dimensional volume projections of fused microPET/CT static images in A431-CCK2R [Tu (+)] and A431-mock [Tu (−)] tumour xenograft-bearing BALB/c nude mice 1 and 2 h p.i. of the ^68^Ga-labelled conjugates. [Ki: kidneys]

**Figure 5:**
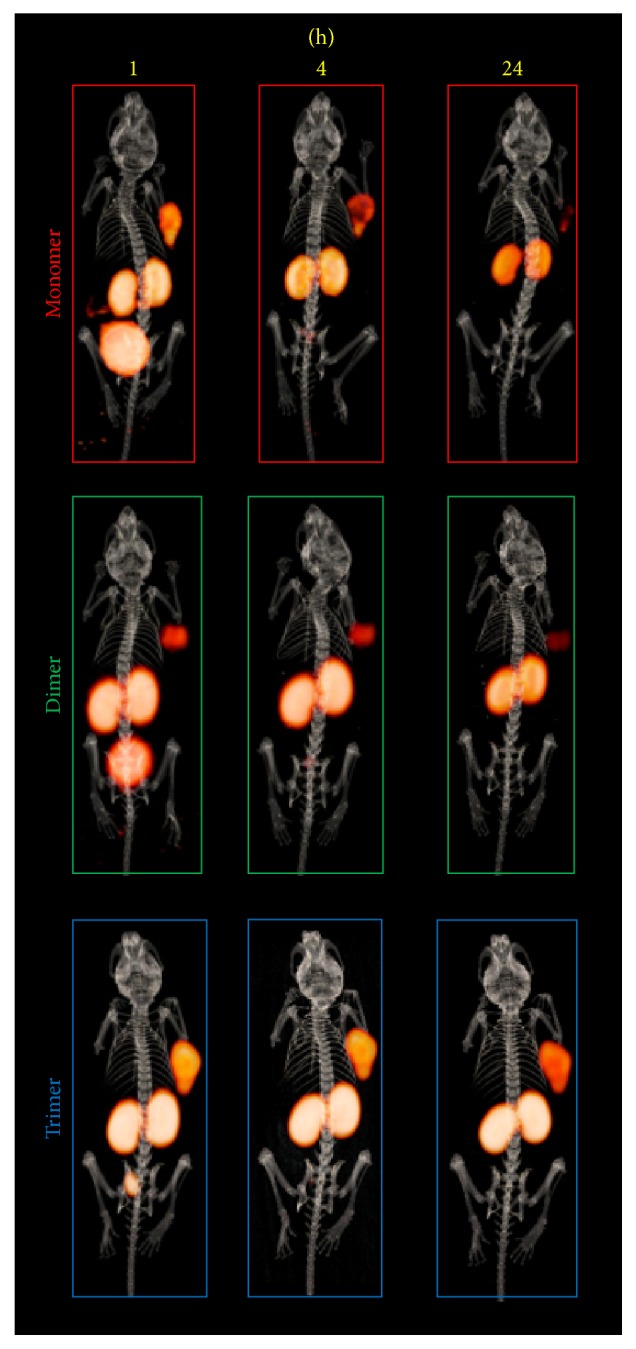
Animal imaging studies of ^89^Zr-labelled conjugates in A431-CCK2R/A431-mock tumour xenografted BALB/C nude mice; 3D volume rendered projections of fused static microPET/CT images.

Investigations on the* in vivo* stability are shown in Figure 3. RP-HPLC analysis of the corresponding blood samples showed increasing amount of intact radioligand to be found in following order trimer > dimer > monomer, indicating that multimerization is accompanied with increased metabolic stability. The results of the* ex vivo* biodistribution studies in double tumour xenografted nude mice are summarized in Table 2 and corresponding tumour-to-organ ratios are presented in Table 3. Mono- and dimeric bioconjugates radiolabelled with gallium-68 were rapidly cleared from the bloodstream and showed highly specific tumour targeting properties as the uptake in nontargeted tissue was very low, except kidneys, 1 h after administration of the radiotracers. Furthermore, the dimer showed significantly (*P* < 0.005) increased tumour uptake compared to the monomer but also higher accumulation in renal tissue. However, the corresponding tumour-to-organ ratios revealed no significant difference in most of the organs. In contrast, the ^68^Ga-trimer cleared slowly from the body and showed higher blood level 1 h p.i. This was substantiated as the uptake in malignant tissue was comparable to the monomer 1 h p.i. but increased consistently over time accompanied by very high accumulation renal tissue. In addition, also an elevated accumulation in nontargeted tissue was observed for the ^68^Ga-trimer, resulting in significantly lower tumour-to-organ ratios compared to mono- and dimer. In comparison, the respective ^89^Zr-labelled counterparts showed a similar behaviour* in vivo* with somewhat lower tumour uptake 1 h p.i. but consistently higher accumulation in renal tissue. ^89^Zr mono- and dimers revealed a trend towards reduced tumour-to-organ ratios as compared to the ^68^Ga-counterparts. In contrast, over time the ^89^Zr-trimer showed a faster elimination from blood and lower unspecific tissue uptake. This resulted in higher tumour-to-organ ratios compared to the ^68^Ga-labelled counterpart especially 4 h p.i. and may be attributed to the increased hydrophilic character of this radiotracer. Small animal PET/CT imaging studies confirmed these findings and the results for ^68^Ga-labelled conjugates are presented in Figure 4 whereas imaging of the ^89^Zr-labelled counterparts is shown in Figure 5. The predominant accumulation of radioactivity found in the kidneys, related to elimination via renal pathway accompanied by tubular reabsorption, was in good agreement with the results of our biodistribution studies and clearly showed that kidney uptake is increased with the grade of multimerization. Furthermore, imaging showed highly specific targeting properties for all conjugates as CCK2 related malignancies were clearly visualized already 1 h after injection without major differences between the bioconjugates. However, imaging at 2 h p.i. for ^68^Ga-labelled and that at 24 h p.i for ^89^Zr-labelled trivalent probe pronounce the increased uptake in CCK2R expressing tissue.

## 4. Discussion

The cyclic siderophore based chelator FSC enables a straightforward multistep synthesis of multivalent imaging probes. Three amines attached to the chelating scaffold of FSC can be utilized for further modifications whereas DOTA, for example, offers only one binding site for conjugation of targeting probes. This results in a different but more symmetrical architecture of FSC-related multivalent bioconjugates. Asymmetric tracer design might not be critical in case of small peptides for targeted imaging but can become an issue in case of multimerization of larger biomolecules (e.g., engineered scaffold proteins, ESP) as the asymmetry can result in less flexibility or increased sterically hindrance at the target interaction site. The suitability for FSC as chelating scaffold for ESP has recently been shown although acetylation of two amines was conducted to design a monovalent affibody construct [18]. Furthermore, FSC can be labelled with gallium-68 and zirconium-89 at RT with sufficient complex stability (Table S1), which is beneficial in case of heat-sensitive molecules. This is also of particular interest for CCK2R targeting peptide derivatives containing methionine as previous studies have shown that heating related oxidation is accompanied by reduced receptor affinity [19]. In contrast, DOTA-conjugates have to be heated for efficient radiolabelling with gallium-68 and in case of oxidation sensitivity of the targeting probe additives (e.g., ascorbic acid) are needed to improve radiochemical purity. Both ^68^Ga and ^89^Zr FSC-conjugates showed* high in vivo* stability; there was also no significant difference in bone uptake between ^68^Ga and ^89^Zr counterparts, indicating insignificant in vivo release of ^89^Zr, confirming recent findings [15].

Overall FSC-based imaging probes showed high CCK2R binding affinity (Figure 1) with values in the low nanomolar range. The divalent probe showing higher affinity is quite consistent with previous reports on ^111^In-MGD5 [11]. Interestingly, increasing the valency from mono- to trimeric constructs, no increase in binding affinity was achieved.

All FSC-based conjugates showed highly specific receptor targeting as demonstrated* in vitro* (Figure 2) and also* in vivo* imaging (Figures 4 and 5) confirmed these findings. Multimerization is accompanied by decreased hydrophilicity and increased binding to serum proteins (Table 2) leading to slower pharmacokinetics* in vivo*, especially for the trimer. Furthermore, kidney retention was considerably increased by the grade of multimerization (Table 3). This is substantiated by the faster blood clearance of ^89^Zr-labelled counterparts due to higher hydrophilicity but increased kidney uptake which may be related to the additional charge introduced as the hexadentate chelator FSC only compensates three of four positive charges of Zr^4+^. Overall, high kidney retention might be critical in case of therapeutic use but is tolerable for diagnostic applications of radiopharmaceuticals [20].

Interestingly, improvement due to multimerization was more pronounced at later time points. While internalization data revealed higher cell uptake at 1 h for the dimer over the trimer, at later time points the trimer showed higher internalization rates. This phenomenon was also seen* in vivo* where the dimer revealed a higher tumour uptake at 1 h p.i., whereas the uptake increased substantially after 2 and 4 h p.i for the trimer exceeding the values of the dimer at 1 h. p.i.. This* in vivo* effects may be explained by a slower target accumulation of the trimer but also by the higher protein binding and slow blood clearance that may act as depot to prolong the tracer concentration at the tumour site, improving imaging contrast over time (Table 3). Furthermore, the improved metabolic stability (Figure 3) of multimeric radioligands targeting CCK2R expression, which has been shown in this study for the first time, also may result in the formation of rebinding metabolites enhancing the imaging contrast over time. This hypothesis is substantiated by the slow tumour washout particularly observed for ^89^Zr-trimer at 24 h p.i (Figure 5) making imaging at late timepoints with multivalent peptides tracers radiolabelled with zirconium-89 not per se uninteresting. Additionally, improving metabolic stability by this multivalency-approach might also be highly interesting for non-radiopharmaceutical-based applications such as therapeutic peptides but definitely warrants further investigations regarding formation of metabolites and their target interaction ability.

## 5. Conclusion

In this study, novel mono- and multimeric bioconjugates utilizing FSC for radiolabelling with gallium-68 and zirconium-89 for PET applications targeting CCK2R expression were synthesized and evaluated for the first time. The resulting imaging probes showed highly specific receptor targeting characteristics which were only partly improved in terms of binding affinity and* in vivo* targeting by the grade of multimerization. However, the higher metabolic stability and improved target retention* in vivo* of multivalent conjugates warrant further investigations on the formation of metabolites with the retained receptor binding ability. Overall this study established FSC as a promising scaffold for the development of mono- and multimeric targeted bioconjugates for molecular imaging with PET.

## Figures and Tables

**Table 1:** Distribution coefficient and protein binding of mono- and multimeric conjugates radiolabelled with gallium-68 and zirconium-89.

		^68^Ga-labelled			^89^Zr-labelled		
		Monomer	Dimer	Trimer	Monomer	Dimer	Trimer
Distribution coefficient	log⁡*D* (pH 7.4)	−2.99 ± 0.02	−2.38 ± 0.04	−2.20 ± 0.07	−3.17 ± 0.06	−2.83 ± 0.02	−2.41 ± 0.04
Protein binding (%)	1 h	3.71 ± 0.86	15.94 ± 1.25	40.69 ± 0.93	5.51 ± 0.41	15.86 ± 0.31	31.52 ± 0.19
	2 h	4.16 ± 0.22	18.77 ± 2.93	44.33 ± 1.41	6.48 ± 0.45	20.83 ± 0.15	35.73 ± 0.92
	4 h	3.16 ± 0.49	17.86 ± 0.32	48.32 ± 1.71	7.28 ± 0.40	26.75 ± 0.49	41.39 ± 2.81

Data are presented as mean ± SD (*n* = 3).

**Table 2:** *Ex vivo* biodistribution studies of ^68^Ga-labelled mono- and multimers as well as ^89^Zr-labelled counterparts in double tumour (±) xenografted BALB/C nude mice; data are presented as percentage of injected dose per gram tissue (% ID/g); mean (*n* = 4) ± SD.

	^68^Ga-labelled					^89^Zr-labelled				
	Monomer	Dimer	Trimer			Monomer	Dimer	Trimer		
	1 h	1 h	1 h	2 h	4 h	1 h	1 h	1 h	2 h	4 h
Blood	0.35 ± 0.06^ab^	0.53 ± 0.06^cd^	3.70 ± 1.27	2.53 ± 0.67	1.76 ± 0.43	0.30 ± 0.19^ab^	1.17 ± 0.28	1.46 ± 0.32^d^	1.19 ± 0.32^d^	0.75 ± 0.26^d^
Spleen	0.18 ± 0.04^ab^	0.37 ± 0.04^cd^	2.15 ± 0.32^e^	2.89 ± 0.53	2.77 ± 0.78	0.12 ± 0.04^ab^	0.99 ± 0.40	0.79 ± 0.23^d^	0.90 ± 0.25^d^	0.95 ± 0.13^d^
Pancreas	0.29 ± 0.02^ab^	0.48 ± 0.06^cd^	1.37 ± 0.26^e^	1.80 ± 0.31	3.10 ± 1.13	0.15 ± 0.04^abd^	0.95 ± 0.23^c^	1.56 ± 0.44	1.51 ± 0.40	2.34 ± 0.59
Stomach	1.07 ± 0.14^ab^	2.13 ± 0.23^c^	3.17 ± 0.54	3.86 ± 1.36^f^	13.6 ± 4.9	0.42 ± 0.14^abd^	2.99 ± 1.13	3.10 ± 0.97	3.15 ± 0.75	3.50 ± 0.86^d^
Intestine	0.58 ± 0.17^b^	0.44 ± 0.06^c^	1.15 ± 0.28^e^	1.86 ± 0.33	2.48 ± 0.59	0.27 ± 0.10^abd^	0.73 ± 0.25	0.56 ± 0.14^d^	0.44 ± 0.30^d^	0.65 ± 0.07^d^
Kidneys	5.57 ± 0.57^abd^	28.9 ± 3.4^cd^	49.7 ± 10.1	59.6 ± 4.3^f^	172.1 ± 14.8	11.3 ± 2.10^ab^	55.5 ± 10.3	57.7 ± 14.4^e^	96.5 ± 23.6^f^	150.5 ± 18.3
Liver	0.23 ± 0.02^ab^	0.78 ± 0.09^c^	3.71 ± 0.75	4.09 ± 0.85	6.01 ± 2.54	0.16 ± 0.04^abd^	0.83 ± 0.20	0.84 ± 0.21^d^	1.28 ± 0.35^d^	1.71 ± 0.32^d^
Heart	0.17 ± 0.02^ab^	0.31 ± 0.04^cd^	1.60 ± 0.28^e^	2.18 ± 0.31	1.51 ± 0.33^f^	0.14 ± 0.05^ab^	1.01 ± 0.37	0.92 ± 0.21^d^	0.91 ± 0.25^d^	0.91 ± 0.09^d^
Lung	0.42 ± 0.06^ab^	0.59 ± 0.08^cd^	4.47 ± 1.50	5.78 ± 0.92	3.75 ± 0.84^f^	0.31 ± 0.09^ab^	1.16 ± 0.28	1.44 ± 0.39^d^	1.21 ± 0.23^d^	0.84 ± 0.20^d^
Muscle	0.20 ± 0.05^b^	0.30 ± 0.08^cd^	0.75 ± 0.15	0.65 ± 0.14	1.01 ± 0.37	0.13 ± 0.04^ab^	1.51 ± 0.70	0.47 ± 0.18^cd^	0.42 ± 0.07^d^	0.62 ± 0.23
Bone	0.31 ± 0.08^b^	0.60 ± 0.21^cd^	2.03 ± 0.88	2.52 ± 0.79	2.26 ± 0.41	0.42 ± 0.10^ab^	1.36 ± 0.42	1.39 ± 0.65	2.07 ± 0.68	4.41 ± 1.31
A431-CCK2R	4.86 ± 1.00^d^	8.36 ± 0.90^ac^	6.07 ± 1.56	8.73 ± 2.65^e^	14.44 ± 3.40^f^	1.91 ± 0.40	7.69 ± 1.34^ac^	4.28 ± 0.95^b^	8.90 ± 3.10^e^	14.39 ± 3.92
A431-mock	0.30 ± 0.04^b^	0.36 ± 0.22^cd^	2.17 ± 0.70^e^	3.18 ± 0.51	2.16 ± 0.54	0.26 ± 0.05^ab^	0.86 ± 0.22	0.81 ± 0.24^d^	0.93 ± 0.29^d^	0.66 ± 0.06^d^

Statistical analysis was performed using the Student's *t*-test with *P* values indicating significant (*P* < 0.05) difference (a) between mono- and dimer, (b) between mono- and trimer, (c) between di- and trimer, (d) between ^68^Ga-labelled tracers and corresponding ^89^Zr-labelled counterparts, (e) between 1 h and 2 h, and (f) between 2 h and 4 h of radiolabelled trimer.

**Table 3:** Corresponding tumour-to-organ ratios of ^68^Ga- and ^89^Zr-labelled bioconjugates; data are presented as mean ± SD.

	^68^Ga-labelled					^89^Zr-labelled				
	Monomer	Dimer	Trimer			Monomer	Dimer	Trimer		
Ratio T/O	1 h	1 h	1 h	2 h	4 h	1 h	1 h	1 h	2 h	4 h
Blood	14.4 ± 3.6^b^	16.0 ± 3.1^cd^	1.8 ± 0.5	3.6 ± 1.0^e^	8.5 ± 2.1^f^	9.0 ± 4.7^b^	7.4 ± 2.3^c^	3.2 ± 0.8^d^	7.3 ± 0.8^de^	20.1 ± 4.8^df^
Spleen	27.3 ± 6.3^b^	23.1 ± 4.2^cd^	2.8 ± 0.4	3.1 ± 0.7	5.5 ± 1.4^f^	18.7 ± 6.1^b^	10.5 ± 4.5	6.1 ± 1.8^d^	9.7 ± 1.5^de^	14.9 ± 2.5^fe^
Pancreas	16.7 ± 3.0^bd^	17.9 ± 3.2^cd^	4.5 ± 1.0^d^	4.9 ± 1.1	4.7 ± 1.5	13.3 ± 2.9^b^	9.2 ± 3.1^c^	2.7 ± 0.2	6.0 ± 2.0^e^	6.3 ± 1.7
Stomach	4.6 ± 1.1^b^	4.0 ± 0.5^c^	2.0 ± 0.8	2.3 ± 0.3^f^	1.1 ± 0.3	4.9 ± 1.3^b^	3.4 ± 1.1	1.6 ± 0.3	2.8 ± 0.7^e^	4.5 ± 1.9^d^
Intestine	9.4 ± 3.7^b^	19.9 ± 4.9^acd^	5.4 ± 1.1	4.8 ± 1.3	6.0 ± 1.3	7.9 ± 2.3	12.5 ± 3.1	8.4 ± 2.0^d^	15.7 ± 1.5^de^	22.2 ± 5.5^d^
Kidneys	0.86 ± 0.13^abd^	0.30 ± 0.06^cd^	0.13 ± 0.05	0.13 ± 0.02^f^	0.07 ± 0.01	0.17 ± 0.03^b^	0.15 ± 0.06	0.08 ± 0.02	0.09 ± 0.02	0.10 ± 0.03
Liver	21.1 ± 3.1^abd^	11.1 ± 2.4^c^	1.7 ± 0.6	2.1 ± 0.4	2.8 ± 1.0	12.4 ± 2.8^b^	10.3 ± 3.2^c^	5.7 ± 1.1^d^	6.9 ± 0.8^d^	8.8 ± 3.1^d^
Heart	29.5 ± 9.3^bd^	27.2 ± 4.8^cd^	3.8 ± 1.0	4.0 ± 0.8	10.1 ± 3.2^f^	14.8 ± 4.7^b^	9.8 ± 4.8	5.0 ± 1.0	9.7 ± 1.2^de^	15.6 ± 2.7^df^
Lung	11.7 ± 2.1^bd^	14.4 ± 2.3^cd^	1.5 ± 0.4	1.5 ± 0.5	3.9 ± 0.8^f^	6.5 ± 1.6^b^	7.6 ± 2.9^c^	3.3 ± 0.7^d^	7.1 ± 1.5^de^	17.3 ± 2.9^df^
Muscle	20.4 ± 1.8^b^	31.4 ± 13.2^cd^	8.4 ± 2.6	14.5 ± 5.9^e^	13.5 ± 5.4	15.9 ± 4.2^b^	7.9 ± 5.3	8.4 ± 0.9	20.7 ± 6.1^e^	25.4 ± 6.8
Bone	17.1 ± 7.4^bd^	11.6 ± 1.9^c^	2.6 ± 1.0	3.8 ± 1.3	7.2 ± 2.6	4.7 ± 1.3^b^	7.4 ± 1.2^c^	2.2 ± 0.1	4.4 ± 1.9	3.4 ± 0.8
A431-mock	16.3 ± 3.3^bd^	17.9 ± 6.1^c^	2.9 ± 0.4	2.9 ± 0.5	7.0 ± 1.9^f^	7.5 ± 1.8	11.2 ± 2.3	6.3 ± 2.6^d^	10.7 ± 4.3^d^	21.6 ± 5.0^df^

Statistical analysis was performed using the Student's *t*-test with *P* values indicating significant (*P* < 0.05) difference (a) between mono- and dimer, (b) between mono- and trimer, (c) between di- and trimer, (d) between ^68^Ga-labelled tracers and corresponding ^89^Zr-labelled counterparts, (e) between 1 h and 2 h, and (f) between 2 h and 4 h of radiolabelled trimer.

## Abbreviations

FSC: Fusarinine C
MG: Minigastrin
CCK2: Cholecystokinin receptor subtype 2
PET/CT: Positron emission computed tomography
RT: Room temperature
DOTA: 1,4,7,10-Tetraazacyclododecane-1,4,7,10-tetraacetic acid
DTPA: Diethylene triamine pentaacetic acid
PBS: Phosphate buffered saline
BSA: Bovine serum albumin
HEPES: 4-(2-Hydroxyethyl)-1-piperazineethanesulfonic acid buffer
TRIS: Tris-(hydroxymethyl)aminomethane
EDTA: Ethylenediaminetetra acetic acid
RP-HPLC: Reversed-phase high-performance liquid chromatography
TLC: Thin layer chromatography
p.i.: Postinjection.

## Supplementary Materials

Supplementary Materials This section contains a detailed description regarding the synthesis of the conjugates. Furthermore representative RP-HPLC chromatograms of ^68^Ga-labelled as well as radio-ITLC analysis of ^89^Zr-labelled probes are shown in Figure S1. The results of transchelation studies for ^68^Ga- and ^89^Zr-labelled conjugates are presented in Table S1. Figure S1: (A) radio-RP-HPLC chromatograms of ^68^Ga- and (B) radio-ITLC analysis of ^89^Zr-labelled mono- and multimeric conjugates. Table S1: transchelation studies of ^68^Ga and ^89^Zr mono- and multimers; data is expressed as percentage of peptide-associated radioactivity.
