# Supplementary material for: Exploiting the Concept of Multivalency with 68Ga- and 89Zr-Labelled Fusarinine C-Minigastrin Bioconjugates for Targeting CCK2R Expression
Source: Contrast Media Mol Imaging. 2018 Apr 10;2018:3171794. doi: 10.1155/2018/3171794 (PMC5914118; doi:10.1155/2018/3171794)
Supplement: Supplementary Materials — This section contains a detailed description regarding the synthesis of the conjugates. Furthermore representative RP-HPLC chromatograms of 68Ga-labelled as well as radio-ITLC analysis of 89Zr-labelled probes are shown in Figure S1. The results of transchelation studies for 68Ga- and 89Zr-labelled conjugates are presented in Table S1. Figure S1: (A) radio-RP-HPLC chromatograms of 68Ga- and (B) radio-ITLC analysis of 89Zr-labelled mono- and multimeric conjugates. Table S1: transchelation studies of 68Ga and 89Zr mono- and multimers; data is expressed as percentage of peptide-associated radioactivity. [file 3171794.f1.docx]

Supplementary Materials for

Exploiting the concept of multivalency with ^68^Ga- and ^89^Zr-labelled Fusarinine C-minigastrin bioconjugates for targeting CCK2R expression

Dominik Summer^1^, Christine Rangger^1^,Maximilian Klingler^1^, Peter Laverman^2^, Gerben M. Franssen^2^, Elisabeth Lechner^3^, Thomas Orasch^3^, Hubertus Haas^3^, Elisabeth von Guggenberg^1^ and Clemens Decristoforo^1,*^

^1^Department of Nuclear Medicine, Medical University Innsbruck, Anichstrasse 35, A-6020 Innsbruck, Austria; ^2^Department of Radiology & Nuclear Medicine, Radboud University Medical Center, Geert Grooteplein Zuid 10, 6525 GA Nijmegen, The Netherlands; ^3^Division of Molecular Biology, Medical University Innsbruck, Innrain 80/82, A-6020 Innsbruck, Austria

***Corresponding Author**

E-mail: Clemens.Decristoforo@i-med.ac.at; Tel: +4351250480951; Fax: +435125046780951

**Precursor synthesis.**

**Peptide synthesis. [3-MP^0^-D-Glu^1^,desGlu^2-6^]-Ala-Tyr-Gly-Trp-Met-Asp-Phe-NH_2_ [MG11-SH].** The synthesis of the MG analogue was carried out following straight forward solid-phase peptide synthesis (SPPS) as previously described[1]. Rink amide MBHA resin (100–200 mesh, 62.5 µmol), *N*-terminal 9-Fluorenylmethoxycarbonyl (Fmoc) and side-chain protected amino acids (Boc-Trp, OtBu for Asp, Tyr and D-Glu) were purchased from Novabiochem (La Jolla, CA, USA). O-(7-azabenzotriazol-1-yl)-1,1,3,3-tetramethyluronium-hexafluorophosphate (HATU) and 1-Hydroxy-7-azabenzotriazole (HOAt) both from GenScript Corporation (Piscataway, NJ, USA) were used to activate the amino acids for conjugation and a threefold molar excess (187.5 μmol) was used to ensure coupling efficiency. For the introduction of a thiol functionality (3-mercaptopropionyl, 3-MP^0^) in the peptide 3-trithylsulfonylpropionic acid (Bachem, Bubendorf, Switzerland) was coupled in a final step. Simultaneous side chain deprotection and resin cleavage was carried out under acidic conditions (TFA/Triisopropylsilane/H_2_O (v/v/v; 95/2.5/2.5)). After precipitation of the crude product in ice-cold diethyl ether the peptide was dissolved in methanol and was purified by preparative RP-HPLC (gradient B; t_R_ = 21.6 min) to give a white powder after freeze-drying. MG11-SH 37.6 mg [34.0 µmol, 54%]; analytical data: RP-HPLC t_R_ = 12.8 min; m/z [M+Na]+ = 1128.23 [C_51_H_64_N_10_O_14_S_2_; exact mass: 1105.24 (calculated)].

**[Fe]Fusarinine C ([Fe]FSC).** FSC was obtained from fungal culture using a method described by Schrettl and co-workers[2]. Seeding an *Aspergillus fumigatus* mutant strain (ΔsidG), that lacks conversion of FSC to triacetylfusarinine C, in 200 mL iron-deficient *Aspergillus* minimal media (1x10^6^ spores/mL) and incubating for 28 hours at 37°C and 200 rpm shaking resulted in secretion of the siderophore FSC by the fungal strain into the culture supernatant. After filtering off the biomass and adding 10 mL FeSO_4_ (100 mM) for iron saturation of FSC, the filtrate was subsequently loaded to a column containing Amberlite XAD18 beads (Dow Chemical Company, Philadelphia, PA, USA). [Fe]FSC was eluted from the column using methanol and after evaporation of the organic solvent ~ 100 mg of product was obtained as red brown coloured solid in > 90% purity. Analytical data: RP-HPLC t_R_ = 8.1 min; m/z [M+H]+ = 780.86 [C_33_H_51_FeN_6_O_12_; exact mass: 779.63 (calculated)].

**Acetylation of [Fe]Fusarinine C.** [Fe]FSC (25 mg, 32 µmol) dissolved in 500 µL dry methanol was reacted with 10 µL of acetic anhydride for 5 min at room temperature (RT) under vigorously stirring. The resulting mixture of mono-, di- and triacetylfusarinine C was immediately purified *via* preparative RP-HPLC using gradient A to obtain *N*-acetylfusarinine C (mAc[Fe]FSC, t_R_ = 17.8 min) and *N,N´*-diacetylfusarinine C (dAc[Fe]FSC, t_R_ = 20.3 min) in high purity (> 95%) followed by lyophilization.

mAc[Fe]FSC 11.9 mg [14.5 µmol, 45%]; analytical data: RP-HPLC t_R_ = 8.4 min; m/z [M+H]+ = 822.90 [C_35_H_53_FeN_6_O_13_; exact mass: 821.67 (calculated)].

dAc[Fe]FSC 8.2 mg [9.5 µmol, 30%]; analytical data: RP-HPLC t_R_ = 8.8 min; m/z [M+H]+ = 864.95 [C_37_H_55_FeN_6_O_14_; exact mass: 863.71 (calculated)].

**Functionalization of FSC and acetylated FSC derivatives with maleimide linker.** [Fe]FSC (6.4 µmol), mAc[Fe]FSC (6.0 µmol) and dAc[Fe]FSC (5.8 µmol) were each dissolved in 500 µL dry dimethylformamide (DMF) and 3-(maleimido)propionic acid (mal) *N*-hydroxysuccinimide ester (Sigma-Aldrich Handels GmbH, Vienna, Austria) was added in 2-4 fold molar excess. After adding 1-3 equivalent of Hünig’s base the reactions were completed within 10 min at ambient temperature (analytical RP-HPLC: [Fe]FSC-(*N,N´,N´´*-(3-maleinimidopropionyl))_3_ = **[Fe]FSC-mal_3_** t_R_ = 12.2 min; mAc[Fe]FSC-(*N´,N´´*-(3-maleinimidopropionyl))_2_ = **mAc[Fe]FSC-mal_2_** t_R_ = 11.1 min; dAc[Fe]FSC-(*N´´*-(3-maleinimidopropionyl)) = **dAc[Fe]FSC-mal** t_R_ = 10.4 min). Subsequently the organic solvent was evaporated, 5 mL of aqueous Na_2_EDTA (100 mM) was added to each vial and stirring was continued overnight. When analytical RP-HPLC analysis confirmed complete removal of iron the products were isolated by preparative RP-HPLC [gradient B: FSC-mal_3_ t_R_ = 25.3 min; mAcFSC-mal_2_ t_R_ = 22.8 min; dAcFSC-mal t_R_ = 19.6 min] directly from the reaction solutions to give colourless solids after lyophilisation in high purity (> 98%).

FSC-mal_3_: 5.5 mg [4.7 µmol, 73%]; analytical data: RP-HPLC t_R_ = 12.6 min; m/z [M+Na]+ = 1203.27 [C_54_H_69_N_9_O_21_; exact mass: 1180.17 (calculated)].

mAcFSC-mal_2_: 5.1 mg [4.8 µmol, 78%]; analytical data: RP-HPLC t_R_ = 11.4 min; m/z [M+H]+ = 1072.21 [C_49_H_66_N_8_O_19_; exact mass: 1071.09 (calculated)].

dAcFSC-mal: 4.8 mg [5.0 µmol, 86%]; analytical data: RP-HPLC t_R_ = 10.8 min; m/z [M+H]+ = 963.03 [C_44_H_63_N_7_O_17_; exact mass: 962.01 (calculated)].

**Conjugation of targeting vector(s).** For site specific conjugation of targeting peptide FSC-mal_3_ (0.85 µmol), mAcFSC-mal_2_ (0.93 µmol) and dAcFSC-mal (1.04 µmol) were each dissolved in phosphate buffered saline (PBS, pH 7.2) and the thiolated MG analogue (MG11-SH) was added in 1.5-4 fold molar excess. Conjugation was completed after 2 h continuous stirring at ambient temperature and the products were freeze-dried after isolation in high purity (>95%) *via* preparative RP-HPLC using gradient C (FSC-(mal-Met)_3_ t_R_ = 22.9 min; mAcFSC-(mal-Met)_2_ t_R_ = 20.6 min; dAcFSC-mal-Met t_R_ = 18.7 min).

FSC-(mal-MG11)_3_ [=Trimer] 2.4 mg [0.53 µmol, 63%]; analytical data: RP-HPLC t_R_ = 16.9 min; m/z [M+H]+ = 4496.65 [C_207_H_261_N_39_O_63_S_6_; exact mass: 4495.90 (calculated)].

mAcFSC-(mal-MG11)_2_ [=Dimer] 2.2 mg [0.67 µmol, 72%]; analytical data: RP-HPLC t_R_ = 15.7 min; m/z [M+Na]+ = 3304.66 [C_151_H_194_N_28_O_47_S_4_; exact mass: 3281.58 (calculated)].

dAcFSC-mal-MG11 [=Monomer] 1.8 mg [0.87 µmol, 84%]; analytical data: RP-HPLC t_R_ = 13.6 min; m/z [M+Na]+ = 2090.99 [C_95_H_127_N_17_O_31_S_2_; exact mass: 2067.25 (calculated)].

**References**

[1] Summer D, Grossrubatscher L, Petrik M, Michalcikova T, Novy Z, Rangger C, et al. Developing Targeted Hybrid Imaging Probes by Chelator Scaffolding. Bioconjug Chem 2017;28:1722–33. doi:10.1021/acs.bioconjchem.7b00182.

[2] Schrettl M, Bignell E, Kragl C, Sabiha Y, Loss O, Eisendle M, et al. Distinct roles for intra- and extracellular siderophores during Aspergillus fumigatus infection. PLoS Pathog 2007;3:1195–207. doi:10.1371/journal.ppat.0030128.


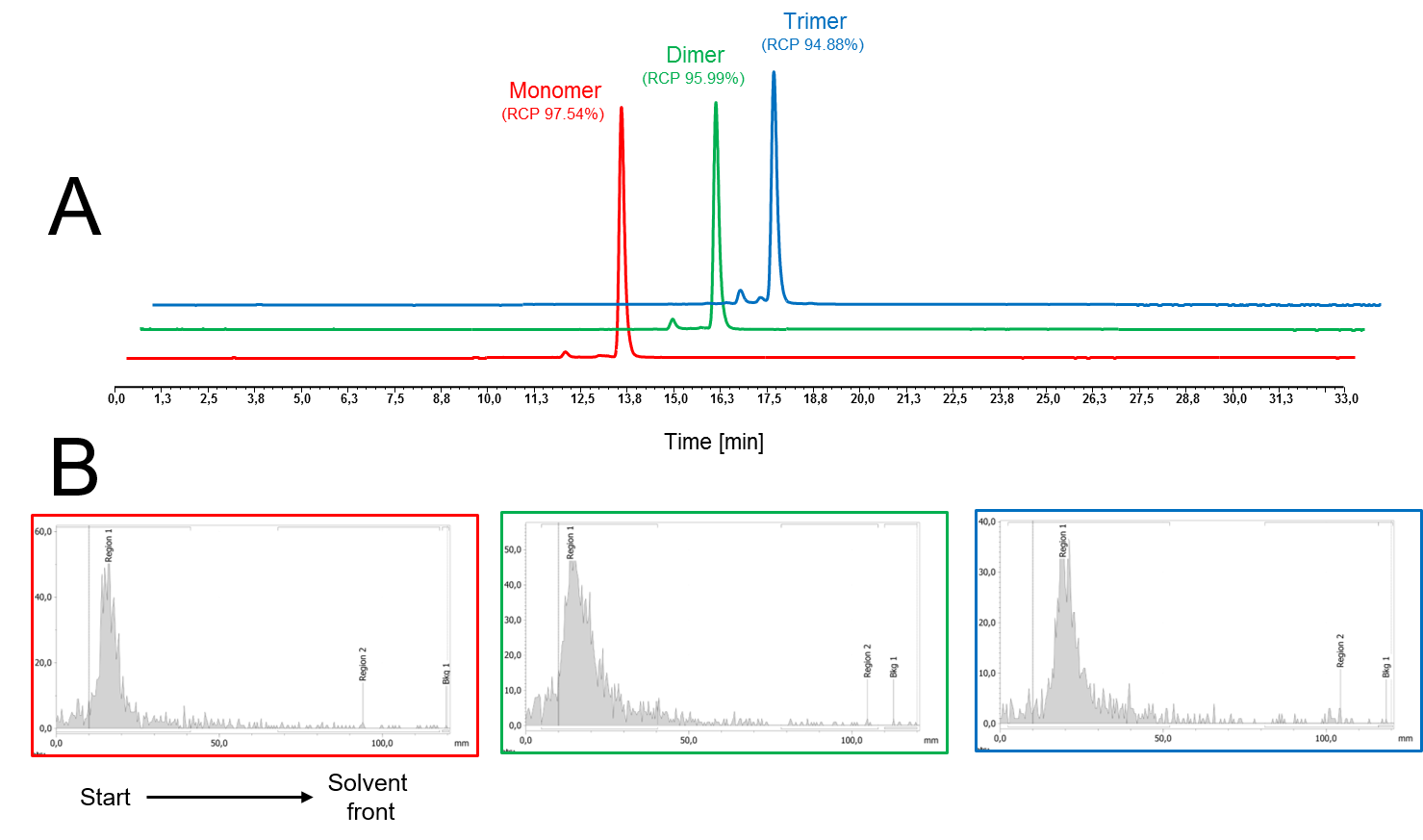


Figure S1: (A) *radio*-RP-HPLC chromatograms of ^68^Ga- and (B) *radio*-ITLC analysis of ^89^Zr-labelled mono and multimeric conjugates

**Table S1:** Trans-chelation studies of ^68^Ga- and ^89^Zr-mono- and multimers; data is expressed as percentage of peptide-associated radioactivity

|  |  | ^68^Ga-labelled | | | ^89^Zr-labelled | | |
| --- | --- | --- | --- | --- | --- | --- | --- |
|  | Incubation Time | Monomer | Dimer | Trimer | Monomer | Dimer | Trimer |
| PBS | 1 h | 99.9 ± 0.1 | 99.9 ± 0.1 | 99.8 ± 0.1 | 99.7 ± 0.1 | 99.5 ± 0.1 | 99.8 ± 0.1 |
|  | 2 h | 99.9 ± 0.1 | 99.9 ± 0.1 | 99.9 ± 0.1 | 99.5 ± 0.1 | 99.5 ± 0.1 | 99.7 ± 0.1 |
|  | 4 h | 99.8 ± 0.2 | 99.9 ± 0.1 | 99.9 ± 0.1 | 99.3 ± 0.1 | 99.5 ± 0.1 | 99.6 ± 0.1 |
|  | 7 d | - | - | - | 99.6 ± 0.1 | 99.8 ± 0.1 | 99.9 ± 0.1 |
| EDTA | 1 h | 98.3 ± 0.1 | 98.2 ± 0.1 | 98.5 ± 0.1 | 99.5 ± 0.1 | 99.4 ± 0.1 | 99.5 ± 0.1 |
|  | 2 h | 97.9 ± 0.1 | 97.7 ± 0.1 | 97.8 ± 0.1 | 99.0 ± 0.1 | 99.2 ± 0.1 | 99.3 ± 0.1 |
|  | 4 h | 97.0 ± 0.5 | 96.3 ± 0.3 | 96.7 ± 0.1 | 98.5 ± 0.1 | 98.7 ± 0.1 | 95.8 ± 0.3 |
|  | 7 d | - | - | - | 85.8 ± 0.2 | 79.3 ± 0.9 | 83.4 ± 0.8 |
| DTPA | 1 h | 99.2 ± 0.1 | 98.9 ± 0.1 | 99.0 ± 0.1 | 99.0 ± 0.1 | 99.0 ± 0.1 | 99.2 ± 0.1 |
|  | 2 h | 99.0 ± 0.1 | 98.8 ± 0.1 | 99.0 ± 0.2 | 99.0 ± 0.2 | 98.9 ± 0.1 | 98.9 ± 0.2 |
|  | 4 h | 98.5 ± 0.1 | 98.6 ± 0.1 | 98.7 ± 0.1 | 98.5 ± 0.2 | 98.8 ± 0.1 | 98.8 ± 0.1 |
|  | 7 d | - | - | - | 99.1 ± 0.1 | 99.3 ± 0.1 | 99.7 ± 0.1 |
| FeCl_3_ | 1 h | 98.0 ± 0.3 | 99.2 ± 0.1 | 98.9 ± 0.2 | 98.7 ± 0.1 | 98.7 ± 0.1 | 98.7 ± 0.1 |
|  | 2 h | 97.3 ± 0.1 | 99.1 ± 0.1 | 97.9 ± 0.3 | 98.7 ± 0.1 | 98.6 ± 0.2 | 98.4 ± 0.1 |
|  | 4 h | 95.6 ± 0.9 | 98.6 ± 0.2 | 97.2 ± 0.4 | 98.1 ± 0.4 | 98.4 ± 0.1 | 98.3 ± 0.1 |
|  | 7 d | - | - | - | 97.9 ± 0.1 | 98.7 ± 0.1 | 98.9 ± 0.1 |
| Human Serum | 1 h | 99.9 ± 0.1 | 99.6 ± 0.1 | 99.5 ± 0.1 | 98.6 ± 0.2 | 98.7 ± 0.1 | 98.6 ± 0.1 |
|  | 2 h | 99.8 ± 0.1 | 99.6 ± 0.1 | 99.4 ± 0.1 | 98.5 ± 0.1 | 98.7 ± 0.2 | 98.6 ± 0.2 |
|  | 4 h | 99.7 ± 0.1 | 99.7 ± 0.1 | 99.3 ± 0.2 | 98.5 ± 0.1 | 98.6 ± 0.2 | 98.7 ± 0.2 |
|  | 7 d | - | - | - | 96.9 ± 0.1 | 97.8 ± 0.1 | 96.8 ± 0.3 |

Data are presented as average ± maximum error (n=2)
